# Factors of heavy social media use among 13-year-old adolescents on weekdays and weekends

**DOI:** 10.1007/s12519-023-00690-1

**Published:** 2023-02-20

**Authors:** Yue-Yue You, Junwen Yang-Huang, Hein Raat, Amy van Grieken

**Affiliations:** 1 The Generation R Study Group, Erasmus Medical Center, Dr. Molewaterplein 40, 3015 GD Rotterdam, the Netherlands; 2 Department of Public Health, Erasmus Medical Center, Dr. Molewaterplein 40, 3015 GD Rotterdam, the Netherlands

**Keywords:** School-aged children, Screen-based behaviors, Social–cultural home environment, Social media use, Socioeconomic position

## Abstract

**Background:**

Few studies have investigated which factors were related to adolescents’ social media use. This study aimed to evaluate which factors were associated with heavy social media use on weekdays and weekends among 13-year-old adolescents.

**Methods:**

We analyzed data from 3727 children from the Generation R Study, a population-based cohort study in the Netherlands. Associations of demographic factors (child age, sex, ethnic background, and family situation), socioeconomic position (parental educational level, parental employment status, and net household income), screen-based behaviors (computer playing and TV viewing), and the home environment (communication, supervision, and restriction) with adolescents’ heavy social media use (≥ 2 hours/day) were assessed separately on weekdays and weekends. Multivariate logistic regression analysis was applied.

**Results:**

The prevalence of heavy social media use was 37.7% on a weekday and 59.6% on a weekend day. Being a girl, living in a one-parent family, and more time spent playing on the computer were associated with heavy social media use on weekdays and weekends (all *P* < 0.05). Low socioeconomic position adolescents (low parental educational level and low household income) were more likely to show heavy social media use only on weekends (all *P* < 0.05). Children whose social media use was restricted by parents on weekdays or children whose social media use was supervised by parents on weekends had lower odds of heavy social media use (all *P* < 0.05).

**Conclusions:**

Being a girl, living in a one-parent family, or having a longer computer playing time were associated with heavy social media use on weekdays and weekends. More studies are needed to understand the factors associated with heavy social media use and the impact of heavy social media use on child health.

**Supplementary Information:**

The online version contains supplementary material available at 10.1007/s12519-023-00690-1.

## Introduction

In recent years, social media has achieved notable popularity worldwide, and adolescents are more likely than any other age group to use social media [1]. For example, a recent study conducted by six European countries among 14- to 17-year-old children showed that 69.5% of European adolescents use social media daily, with 38.8% reporting social media use for more than 2 hours per day [2]. Previous studies have indicated the beneficial effects of social media use among adolescents, including learning to express themselves and new skills [3, 4]. However, a systematic review reported that heavy social media use might cause negative implications on adolescents’ mental health and well-being, given the unprecedented speed at which social media has become ubiquitous [5]. For example, Abi-Jaoude et al. reported that heavy social media use could increase mental distress, self-injury behavior and suicidality among youth; moreover, there is a dose‒response relationship [6]. Therefore, identifying the factors of heavy social media use may help develop effective prevention programs and provide guidelines for healthy social media use.

Previous studies have explored several factors of social media use among adolescents, focusing mainly on two domains: demographic factors and socioeconomic position (SEP); however, the results are inconsistent in different countries [7–11]. For example, Blackwell et al. found no association between sex and time spent on social media among children in the US, but Spilkova et al. reported that boys exhibited half the likelihood of ≥ 2 hours of social media use than girls among Czech adolescents [7, 8]. In addition, a study conducted by the Pew Research Center in the US found that adolescents from middle and upper SEP families had higher daily social media use than their counterparts [9]. In contrast, Hugues et al. found no association between SEP and time spent on social media among children in Canada [10]. The inconsistent findings on the associations of demographic factors and SEP with heavy social media use suggest that more research is needed.

Furthermore, several other domains, such as screen-based behaviors or home environments, may contribute to social media use among adolescents. In the screen-based behaviors domain, Rideout et al. pointed out that media multitasking is increasingly common in the younger generation; among adolescents, the percentage of media multitasking was 60% in 2015 [12]. In addition, Ettinger et al. reported that most multitasking involves combinations of social media or video games among adolescents [13]. In the home environment domain, social-ecological theory suggests that the environment interacts with health behaviors and outcomes [14]. For example, parents use several mechanisms to regulate or monitor their children’s behaviors, such as establishing rules, which may help reduce heavy social media use. However, studies on the association between other screen-based behaviors, home environment and heavy social media use among adolescents are relatively scarce [15, 16].

As adolescents spend more time on their phones or other devices on weekends than on weekdays (as they are at school during the day), we examined social media use on weekdays and weekends separately in this paper [17]. Most research investigating factors of heavy social media use uses overall days of measurement [8]. It could limit the identification of factors specific to weekday or weekend heavy social media use, which could be important to successfully prevent heavy social media use.

The current study aimed to identify which factors were associated with heavy social media use and whether factors differed between weekdays and weekends among adolescents aged 13 years. All factors were studied across four domains: demographic factors (child’s age, sex, ethnic background, and family situation), SEP (parental educational level, parental employment status, and household income), other screen-based behaviors (computer playing and TV viewing), and home environment (communication, supervision, and restriction).

## Methods

### Study design and population

The data used in this study come from the Generation R Study. Generation R is a multiethnic population-based cohort from fetal life onward. It has previously been described in detail [18]. Briefly, all women living in Rotterdam, the Netherlands, with an expected delivery date between April 2002 and January 2006, were eligible for participation. Consent for postnatal follow-up was available for 6842 children aged 13 years [19]. Children with missing data on all factors (*n* = 444) and social media (*n* = 2303) were excluded. To avoid clustering of data, the second (*n* = 360) and third children (*n* = 8) of the same mother were excluded, leaving a study population of 3727 participants. The study was conducted in accordance with the guidelines proposed in the Declaration of Helsinki. The Medical Ethics Committee of the Erasmus Medical Center, Rotterdam, approved the study. Written informed consent was obtained from all the participants.

### Measurements

#### Demographic factors

Child age (years), sex (boy, girl), ethnic background, and family situation were used as indicators of demographic factors, which were obtained by parent-reported questionnaires. The child’s ethnic background (Dutch, other Western and non-Western) was based on the parents’ country of birth, which was assessed by a questionnaire when the child was 6 years old. If one of the parents was born outside the Netherlands, this country of birth determined the ethnic background of the child. If both parents were born outside the Netherlands, the mother’s country of birth determined the ethnic background [20]. The family situation was categorized as a two-parent family and a one-parent family and was obtained when the child was 13 years old.

#### Socioeconomic position (SEP)

Maternal and paternal educational level, maternal and paternal employment status, and net household income were used as indicators of SEP, which were obtained by parent-reported questionnaires. Maternal and paternal educational levels were obtained when the child was 6 years old and categorized as follows: low (no education, primary school, lower vocational training, intermediate general school, or 3 years or less general secondary school), middle (more than 3 years general secondary school, intermediate vocational training, first year of higher vocational training, or higher vocational training), and high (university or PhD degree) [21]. Maternal and paternal employment status (paid job, no paid job) and net household income per month (< €2000/month, €2000-€3600/month, > €3600/month) were obtained when the child was 13 years old.

#### Other screen-based behaviors

Computer playing (continuous) and TV viewing (continuous) were used as indicators of other screen-based behaviors, which were obtained by the adolescent self-report questionnaire when the adolescent was 13 years old. Adolescents were asked to report: “How many hours per weekday do you usually spend playing games on a computer? and “How many hours per weekend day do you usually spend playing games on a computer?”. TV viewing time was also obtained by the same method.

#### Home environment

Communication, supervision, and restriction were used as indicators of the home environment, which were obtained by a parent-reported questionnaire when the adolescent was 13 years old. For communication, parents answered the question “I talk to my child about what they see when they are on social media”; for supervision, parents answered the question “I sit at the computer together with my child if they are on social media”; for restriction, parents answered the question “I restrict the time my child is allowed to visit social media”. Response options were never, rarely, sometimes, often, and always. We grouped these options into two categories: never/rarely versus sometimes/often/always.

#### Adolescents’ time spent on social media

Time spent on social media was examined separately on weekdays and weekends since adolescents spend more time on their devices on weekends than on weekdays, as they are at school during the weekdays. Adolescents were asked to report “On average, how many hours per weekday do you usually spend on social media?” and “On average, how many hours per weekend day do you usually spend on social media?”. Based on the previously used definition of heavy social media use, a binary measure was constructed to contrast regular use (< 2 hours/day) against heavy use (≥ 2 hours/day) [22, 23].

### Statistical analysis

#### Data analysis

**Fig. 1:**
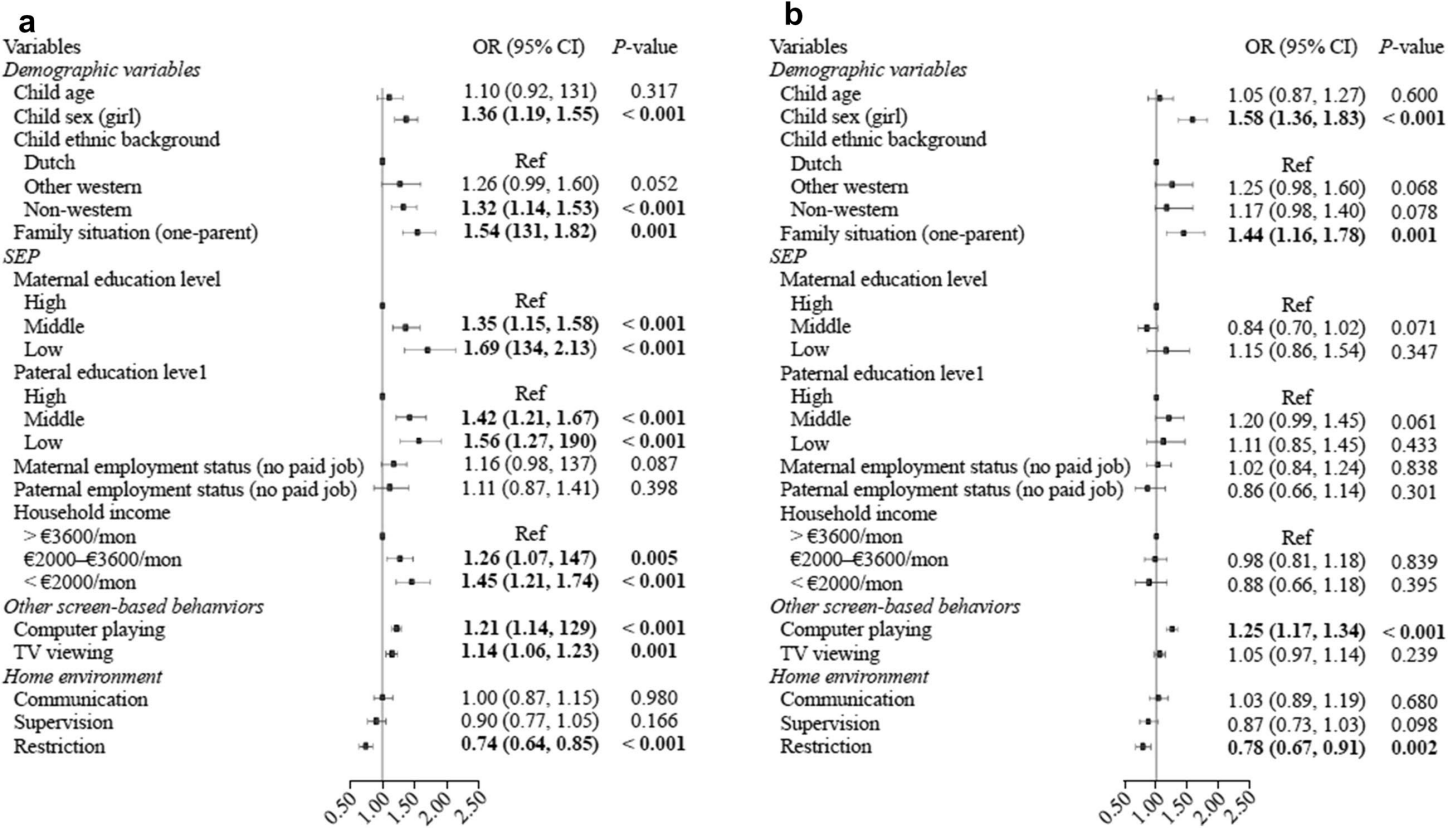
Factors of heavy social media use among 13-year-old adolescents on a weekday. **a** Univariate mode 1; **b** multivariate mode 1. *SEP* socioeconomic position, *CI* confidence interval, *OR* odds ratio

**Fig. 2:**
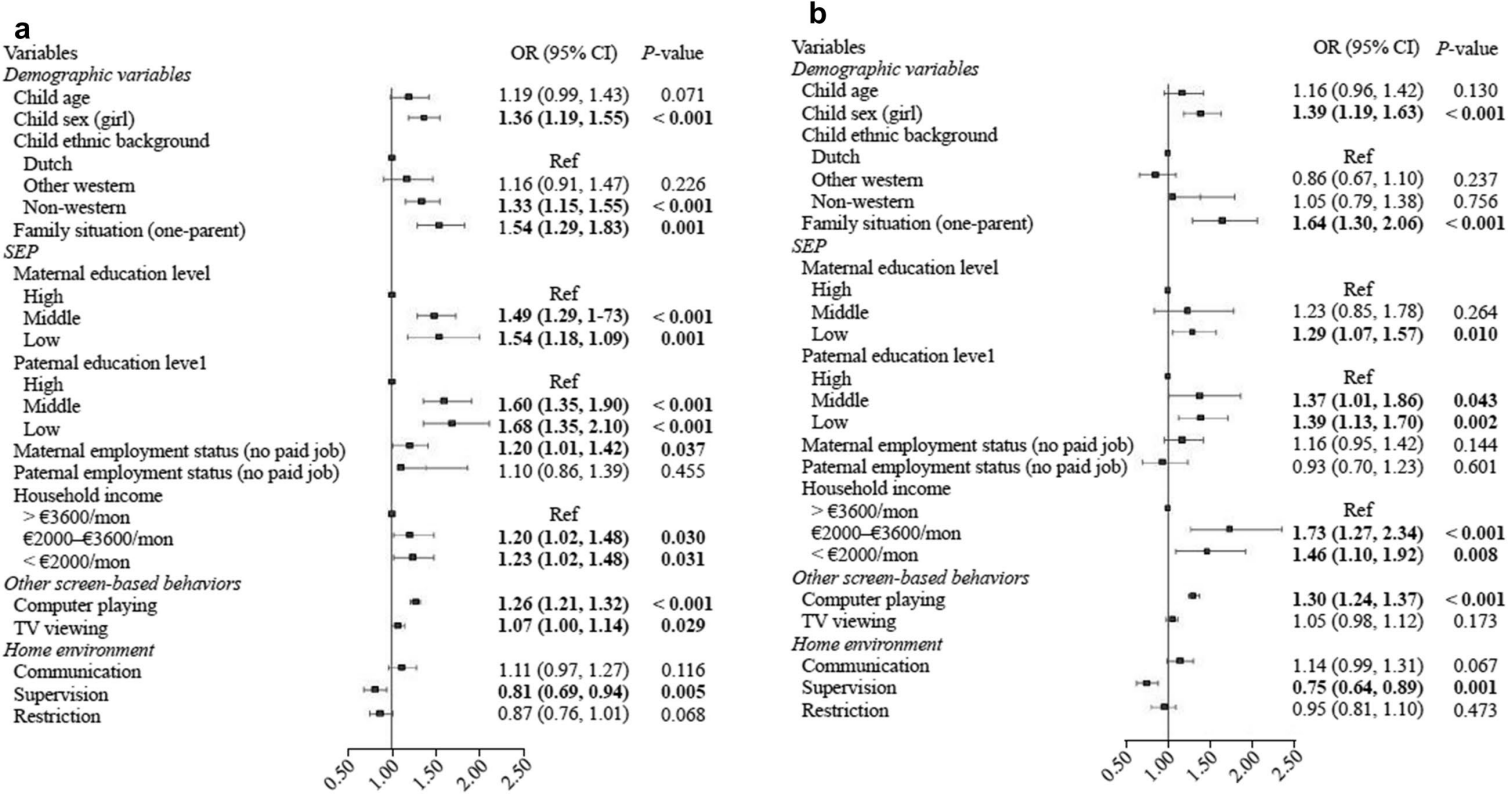
Factors of heavy social media use among 13-year-old adolescents on a weekend day. **a** Univariate mode 1; **b** multivariate mode 1. *SEP* socioeconomic position, *CI* confidence interval, *OR* odds ratio

Descriptive analysis was applied to characterize the study population. Next, logistic regression models were constructed to study factors of heavy social media use separately for weekdays and weekends. In model 1, each factor was entered into the model separately (i.e., univariable model). In model 2, all factors were entered into the model together to assess the effect of each factor corrected for the other variables in the model (i.e., multivariable models). The results of all models are also presented as figures (Figs. 1 and 2). The correlations between the independent variables were analyzed using Spearman’s r. The correlation coefficient did not indicate collinearity; therefore, all variables were simultaneously included in the full models.

Multiple imputations by fully conditional specification (FCS MI) were used to address the missing values on all independent variables (ranging from 1.0% to 21.9%, see Table 1), and 10 imputed datasets were generated. Pooled estimates from these 10 imputed datasets were used to report effect estimates [odds ratios (ORs)] and their 95% confidence intervals (CIs). FCS MI is a powerful and statistically valid method for creating imputations in large datasets, which include both categorical and continuous variables. It specifies the multivariable imputation model on a variable-by-variable basis and offers a principled yet flexible method of addressing missing data, which is particularly useful for large datasets with complex data structures [24]. Statistical analyses were performed using IBM SPSS Statistics for Windows, version 25.0. Armonk, NY: IBM Corp. A significance level of *P* < 0.05 was used to indicate significant associations.

**Table 1:** General characteristics of the study population (*n* = 3727). Table is based on a non-imputed dataset. ^a^Communication: parents talk to their child about what the child see when the child is on social media. ^b^Supervision: parents sit at the computer together with their child if their child is on social media. ^c^Restriction: parents restrict the time their child visit social media. ^d^Heavy social media use defined as social media use ≥ 2 hours per day. *SD* standard deviation

Characteristics	Total	Missing, *n* (%)
Demographic variables		
Child age, mean (SD)	13.54 (0.36)	40 (1.1)
Child sex, *n* (%)		
Boy	1810 (48.6)	0
Girl	1917 (51.4)	
Child ethnic background, *n* (%)		
Dutch	2359 (63.9)	38 (1.0)
Other western	326 (8.8)	
Non-western	1004 (27.3)	
Family situation, *n* (%)		
Two-parent family	2922 (80.8)	109 (2.9)
One-parent family	696 (19.2)	
Socioeconomic position		
Maternal education level, *n* (%)		
Low	314 (9.4)	386 (10.4)
Middle	979 (29.3)	
High	2048 (61.3)	
Paternal education level, *n* (%)		
Low	393 (12.7)	642 (17.2)
Middle	792 (25.7)	
High	1900 (61.6)	
Maternal employment status, *n* (%)		
Paid job	2860 (81.2)	207 (5.6)
No paid job	660 (18.8)	
Paternal employment status, *n* (%)		
Paid job	3055 (91.1)	372 (10.0)
No paid job	300 (8.9)	
Household income, *n* (%)		
< €2000/mon	571 (17.1)	386 (10.4)
€2000–€3600/mon	870 (26.0)	
> €3600/mon	1,900 (56.9)	
Other screen time behaviors		
Computer playing (h/d, mean (SD)		
On a weekday	1.15 (1.20)	789 (21.2)
On a weekend day	1.89 (1.61)	815 (21.9)
TV viewing (h/d), mean (SD)		
On a weekday	1.05 (0.98)	798 (21.4)
On a weekend day	1.72 (1.27)	818 (21.9)
Home environment		
Communication^a^, *n* (%)		
Never/rarely	1743 (49.2)	182 (4.9)
Sometimes/often/always	1802 (50.8)	
Supervision^b^, *n* (%)		
Never/rarely	2660 (74.9)	176 (4.7)
Sometimes/often/always	891 (25.1)	
Restriction^c^, *n* (%)		
Never/rarely	1084 (30.8)	213 (5.7)
Sometimes/often/always	2430 (69.2)	
Social media use		
Heavy social media use^d^, *n* (%)		
On a weekday	1404 (37.7)	0
On a weekend day	2223 (59.6)	0

#### Nonresponse analyses

Children with missing data on social media use at 13 years old (*n* = 2303) were compared with those without missing data (*n* = 3727) regarding socio-demographic characteristics using Chi-square tests. Data were often missing for children from a non-Western ethnic background, parents with a low educational level, a low household income, or a mother or father without a paid job (all *P* < 0.05).

#### Sensitivity analyses

To compare the results based on complete case analyses and the imputed dataset, sensitivity analyses were performed. The analyses of the association between factors and heavy social media use on weekdays and weekends were performed on both the non-imputed and imputed datasets. The results from the analyses on the non-imputed dataset are shown in supplemental Tables 1 and 2.

## Results

Table 1 shows the general characteristics of the study population. The mean age of adolescents was 13.54 [standard deviation (SD): 0.36] years, and 48.6% were boys. More than half of the children had a Dutch ethnic background (63.9%), and the most common type of family was a two-parent family (80.8%). In total, 61.3% of the mothers and 61.6% of the fathers had a high educational level. More than half of the children belonged to a family with a high-income household (56.9%). Adolescents spent 1.15 (SD: 1.20) and 1.89 (SD: 1.61) hours on the computer playing on a weekday and a weekend, respectively. The time spent on TV viewing was 1.05 (SD: 0.98) on a weekday and 1.72 (SD: 1.27) on a weekend day. More than half of the parents communicated about (50.8%) and restricted (69.2%) their child’s social media use. The prevalence of heavy social media use was 37.7% (on a weekday) and 59.6% (on a weekend day).

Table 2 and Fig. 1 show the associations between all factors and heavy social media use on a weekday. Being a girl, being from a non-Western ethnic background, living in a one-parent family, having a low family SEP (low parental educational level and low household income), having longer TV viewing and longer computer playing time, and less parental restriction of social media use were significantly associated with heavy social media use (Table 2 model 1; Fig. 1a). After including all factors in the model, associations were observed between sex, family situation, computer playing, restriction, and heavy social media use (Table 2 model 2; Fig. 1b). Girls [odds ratio (OR): 1.58; 95% confidence interval (CI): 1.36, 1.83] or children living in one-parent families (OR: 1.44, 95% CI 1.16, 1.78) were more likely to show heavy social media use than their counterparts. Spending more time on the computer playing on a weekday was associated with heavy social media use (OR: 1.25; 95% CI 1.17, 1.34). Children whose social media use was restricted by parents (OR: 0.78; 95% CI 0.67, 0.91) had lower odds of heavy social media use. The Chi-square by the Omnibus Test was 153.00 (*P* < 0.001), and the Nagelkerke R Squared in the model summary was 7.5% (model 2).

**Table 2:** Factors of heavy social media use on a weekday among 13-year-old adolescents (*n* = 3727). Table is based on imputed dataset. Bold print indicates statistical significance at *P* < 0.05.Values represent odds ratios and 95% confidence intervals derived from (multiple) logistic regression analyses. Model 1: each factor was added to the model separately. Model 2: all factors were added to the model. *OR* odds ratio,* CI* confidence interval

Variables	Model 1		Model 2	
	OR (95% CI)	*P* value	OR (95% CI)	*P* value
Demographic variables				
Child age	1.10 (0.92, 1.31)	0.317	1.05 (0.87, 1.27)	0.600
Child sex				
Boy	1.00		1.00	
Girl	**1.36 (1.19, 1.55)**	** < 0.001**	**1.58 (1.36, 1.83)**	** < 0.001**
Child ethnic background				
Dutch	1.00		1.00	
Other western	1.26 (0.99, 1.60)	0.052	1.25 (0.98, 1.60)	0.068
Non-western	**1.32 (1.14, 1.53)**	** < 0.001**	1.17 (0.98, 1.40)	0.078
Family situation				
Two-parent family	1.00		1.00	
One-parent family	**1.54 (1.31, 1.82)**	** < 0.001**	**1.44 (1.16, 1.78)**	**0.001**
Socioeconomic position				
Maternal education level				
High	1.00		1.00	
Middle	**1.35 (1.15, 1.58)**	** < 0.001**	0.84 (0.70, 1.02)	0.072
Low	**1.69 (1.34, 2.13)**	** < 0.001**	1.15 (0.86, 1.54)	0.347
Paternal education level				
High	1.00		1.00	
Middle	**1.42 (1.21, 1.67)**	** < 0.001**	1.20 (0.99, 1.45)	0.061
Low	**1.56 (1.27, 1.90)**	** < 0.001**	1.11 (0.85, 1.46)	0.433
Maternal employment status				
Paid job	1.00		1.00	
No paid job	1.16 (0.98, 1.37)	0.087	1.02 (0.84, 1.24)	0.838
Paternal employment status				
Paid job	1.00		1.00	
No paid job	1.11 (0.87, 1.41)	0.398	0.86 (0.66, 1.14)	0.301
Household income				
> €3600/mon	1.00		1.00	
€2000–€3600/mon	**1.26 (1.07, 1.47)**	**0.005**	0.98 (0.81, 1.18)	0.839
< €2000/mon	**1.45 (1.21, 1.74)**	** < 0.001**	0.88 (0.66, 1.18)	0.395
Other screen-based behaviors				
Computer playing	**1.21 (1.14, 1.29)**	** < 0.001**	**1.25 (1.17, 1.34)**	** < 0.001**
TV viewing	**1.14 (1.06, 1.23)**	**0.001**	1.05 (0.97, 1.14)	0.239
Home environment				
Communication				
Never/rarely	1.00		1.00	
Sometimes/often/always	1.00 (0.87, 1.15)	0.980	1.03 (0.89, 1.19)	0.680
Supervision				
Never/rarely	1.00		1.00	
Sometimes/often/always	0.90 (0.77, 1.05)	0.166	0.87 (0.73, 1.03)	0.098
Restriction				
Never/rarely	1.00		1.00	
Sometimes/often/always	**0.74 (0.64, 0.85)**	** < 0.001**	**0.78 (0.67, 0.91)**	**0.002**

Table 3 and Fig. 2 show the associations between all factors and heavy social media use on a weekend day. Being a girl, being from a non-Western ethnic background, living in a one-parent family, having a low family SEP (low parental educational level, maternal with unemployment status, and low household income), having longer TV viewing and longer computer playing time, and less parental supervision of social media use were significantly associated with heavy social media use (Table 3 model 1; Fig. 2a). After including all factors in the model, associations between sex, family situation, parental educational level, household income, computer playing, supervision, and heavy social media use were observed (Table 3 model 2; Fig. 2b). Girls (OR: 1.39; 95% CI 1.19, 1.63) or children living in one-parent families (OR: 1.64; 95% CI 1.30, 2.06) were more likely to show heavy social media use than their counterparts. Children from a family with a low SEP, including low maternal educational level (OR: 1.29, 95% C 1.07, 1.57), low paternal educational level (low: OR: 1.39, 95% CI 1.13, 1.70; middle: OR: 1.37, 95% CI 1.01, 1.86) and low household income (low: OR: 1.46, 95% CI 1.10, 1.92; middle: OR: 1.73, 95% CI 1.27, 2.34), had higher odds of heavy social media use than their counterparts. Spending more time playing on a computer on a weekend day was associated with heavy social media use (OR: 1.30; 95% CI 1.24, 1.37). Children whose social media was supervised by parents (OR: 0.75; 95% CI 0.64, 0.89) had lower odds of heavy social media use. The Chi-square by the Omnibus Test was 214.48 (*P* < 0.001), and the Nagelkerke R Squared in the model summary was 12.2% (model 2).

**Table 3:** Factors of heavy social media use on a weekend day among 13-year-old adolescents (*n* = 3727). Table is based on imputed dataset. Bold print indicates statistical significance at *P* < 0.05. Values represent odds ratios and 95% confidence intervals derived from (multiple) logistic regression analyses. Model 1: each factor was added to the model separately. Model 2: all factors were added to the model. *OR* odds ratio*, CI* confidence interval

	Model 1		Model 2	
Varaibles	OR (95% CI)	*P* value	OR (95% CI)	*P* value
Demographic variables				
Child age	1.19 (0.99, 1.43)	0.071	1.16 (0.96, 1.42)	0.130
Child sex				
Boy	1.00		1.00	
Girl	**1.36 (1.19, 1.55)**	** < 0.001**	**1.39 (1.19, 1.63)**	** < 0.001**
Child ethnic background				
Dutch	1.00		1.00	
Other western	1.16 (0.91, 1.47)	0.226	0.86 (0.67, 1.10)	0.237
Non-western	**1.33 (1.15, 1.55)**	** < 0.001**	1.05 (0.79, 1.38)	0.756
Family situation				
Two-parent family	1.00		1.00	
One-parent family	**1.54 (1.29, 1.83)**	** < 0.001**	**1.64 (1.30, 2.06)**	** < 0.001**
Socioeconomic position				
Maternal education level				
High	1.00		1.00	
Middle	**1.49 (1.29, 1.73)**	** < 0.001**	1.23 (0.85, 1.78)	0.264
Low	**1.54 (1.18, 1.99)**	**0.001**	**1.29 (1.07, 1.57)**	**0.010**
Paternal education level				
High	1.00		1.00	
Middle	**1.60 (1.35, 1.90)**	** < 0.001**	**1.37 (1.01, 1.86)**	**0.043**
Low	**1.68 (1.35, 2.10)**	** < 0.001**	**1.39 (1.13, 1.70)**	**0.002**
Maternal employment status				
Paid job	1.00		1.00	
No paid job	**1.20 (1.01, 1.42)**	**0.037**	1.16 (0.95, 1.42)	0.144
Paternal employment status				
Paid job	1.00		1.00	
No paid job	1.10 (0.86, 1.39)	0.455	0.93 (0.70, 1.23)	0.601
Household income				
> €3600/mon	1.00		1.00	
€2000–€3600/mon	**1.20 (1.02, 1.48)**	**0.030**	**1.73 (1.27, 2.34)**	** < 0.001**
< €2000/mon	**1.23 (1.02, 1.48)**	**0.031**	**1.46 (1.10, 1.92)**	**0.008**
Other screen-based behaviors				
Computer playing	**1.26 (1.21, 1.32)**	** < 0.001**	**1.30 (1.24, 1.37)**	** < 0.001**
TV viewing	**1.07 (1.00, 1.14)**	**0.029**	1.05 (0.98, 1.12)	0.173
Home environment				
Communication				
Never/rarely	1.00		1.00	
Sometimes/often/always	1.11 (0.97, 1.27)	0.116	1.14 (0.99, 1.31)	0.067
Supervision				
Never/rarely	1.00		1.00	
Sometimes/often/always	**0.81 (0.69, 0.94)**	**0.005**	**0.75 (0.64, 0.89)**	**0.001**
Restriction				
Never/rarely	1.00		1.00	
Sometimes/often/always	0.87 (0.76, 1.01)	0.068	0.95 (0.81, 1.10)	0.473

Supplementary tables 1 and 2 show the results of (multiple) logistic regression conducted with non-imputed dataset. The results were comparable to those of the main analyses (Tables 2 and 3). The factors associated with heavy social media use on weekdays and weekends were the same compared to the main analyses, although effect estimates (ORs) were smaller. The contribution of each factor is reported in Supplementary Tables 3 and 4.

## Discussion

The present study evaluated factors associated with heavy social media use (≥ 2 hours/day) among 13-year-old children. We studied the associations of demographic factors, socioeconomic position, other screen-based behaviors, and home environment with heavy social media use on weekdays and weekends. The findings showed that the prevalence of heavy social media use among adolescents was 37.7% and 59.6% on weekdays and weekends, respectively. Being a girl, living in a one-parent family, and having longer computer playing time were positively associated with heavy social media use on weekdays and weekends. Interestingly, a low family SEP (low parental educational level and low household income) was significantly associated with heavy social media use only on weekend days. Children whose social media use was restricted by parents on weekdays or children whose social media use was supervised by parents on weekends had lower odds of heavy social media use.

Previous studies have shown a wide range of prevalence rates of heavy social media use, ranging from 25% to 43.6%, potentially due to methodological differences such as small sample sizes [8, 25]. For instance, a nationally representative study in the Czech Republic showed that 25.9% of adolescents spent more than 2 hours on social media per day [8]. In contrast, the prevalence of heavy social media use among adolescents in a province‐wide school‐based survey in Ontario (Canada) was 42.9% [25]. Our study also observed a high prevalence of heavy social media use. Furthermore, compared to previous literature, the current study showed an increase in heavy social media use, especially on weekends (on weekdays: 37.7%; on weekends: 59.6%) [26, 27]. Therefore, our results suggest that preventing heavy social media use on weekends may have the biggest impact on the prevention of heavy social media use.

The association between demographic factors (sex and family situation) and heavy social media use was confirmed in the present study. The results showed that being a girl or children living in one-parent families had higher odds of heavy social media use. Previous studies have shown that girls and boys interact differently with and on social media and consume its content differently [28]. Currently, no definitive explanation for sex differences in heavy social media use is available, and more research on this area is needed.

The results showed that low family SEP (low parental educational level and low-income household) was positively associated with heavy social media use only on weekend days. A possible explanation may be that children may have less free time spent on social media due to a busy school schedule and heavy homework on weekdays, either with a high or a low SEP [29]. However, weekends may provide an opportunity for increasing social media use for children with a low SEP. On weekend days, compared to children from low-income families, children from high-income families are more likely to participate in organized sports that involve multiple expenses (e.g., membership fees, transport fees, and play material fees), such as swimming, cycling, and hockey [30, 31]. These activities may lead to less time for children on social media. In contrast, children from low-income families are more likely not to participate in organized sports and may have more free time to use social media on weekends. In addition, low-educated parents might be less aware of the possible adverse health effects due to heavy social media use, consequently exerting less control over their children’s social media use than highly educated parents [32].

The results also showed that longer computer playing time was associated with heavy social media use on weekdays and weekends. Younger adolescents are most likely to multitask when engaged in media-based behavior [33, 34]. Griffiths et al. reported that teenagers make the most use of social media compared to the general population [35]. They often use social media as a communication tool and utilize its potential for technology-enabled connections when playing computer games [36]. In other words, adolescents use social media to exchange opinions and communicate with their team members when playing games, increasing their social media use. Thus, encouraging adolescents to spend less time on computer playing may be an effective strategy to reduce their social media use. Previous studies have shown that more time spent in health-promoting activities (i.e., physical activity) or face-to-face interactions could effectively reduce adolescent spending time on computer games, which may, in turn, reduce social media use [37].

Furthermore, our results showed that children whose social media use was restricted by parents on weekdays or children whose social media use was supervised by parents on weekends had lower odds of heavy social media use. There are little data available to compare our results, as few studies have examined social–cultural home environment factors of heavy social media use, and fewer have stratified analyses on weekdays and weekends. Previous studies on these factors have mainly focused on adolescents’ traditional media device usage, such as television or computer use [38, 39]. There are a few potential explanations for the differences in associations between social media rules on weekdays and weekends. First, parents might be more available and involved with their children on weekends than on weekdays. For example, Yeung et al. found that parents have more hours of involvement time with their children on a weekend day than on a weekday [40]. Second, parents supervise children’s social media use significantly more on weekends than on weekdays. Overall, these results underscore the critical role of parents in adolescents’ social media use.

Reducing heavy social media use is important for adolescents’ health and well-being. Therefore, youth health care professionals working with families and schools may need guidelines to suggest practical strategies to limit social media use. Moreover, a clear definition of limiting “heavy” use might support professionals in actively promoting less social media use.

### Strengths and limitations

This study’s strength is the large number of adolescents being studied and the collection of data on social media use on weekdays and weekends. In addition, our study assessed parental communication, supervision, and restriction rules. However, the current study has some limitations that should be mentioned. First, our study was cross-sectional and did not permit the establishment of causal relationships. Future work should examine the associations longitudinally. Second, factors of social media use rules were based on parent reports, which is a parent proxy measure. We recommend that future studies measuring these factors use adolescent self-reports together with parent reports. Third, missing information on factors was imputed. Therefore, we used the non-imputed dataset to explore which factors were associated with heavy social media use on weekdays and weekends. All results were comparable to the imputed dataset, suggesting minimal bias. Despite these limitations, our study offers important insights and implications for professionals in developing interventions to promote healthy social media use among school-aged children.

In conclusion, the prevalence of heavy social media use among 13-year-old children was 37.7% and 59.6% during week and weekends, respectively. A low family socioeconomic position was associated with heavy social media use only on weekends. Longer computer playing time was associated with heavy social media use. Children whose social media use was restricted or supervised by parents had lower odds of heavy social media use. These results may help professionals provide advice to families and support intervention development that can promote healthy social media use among children and adolescents.

## Supplementary Information

Below is the link to the electronic supplementary material.

Supplementary file1 (DOCX 22 KB)

## Data Availability

The datasets generated and/or analyzed during the current study are not publicly available due to individual privacy consideration, but are available from the data managers (datamanagementgenr@erasmusmc.nl) and Director Generation R, Vincent Jaddoe (v.jaddoe@erasmusmc.nl) after a written agreement about the use of the data made via the Technology Transfer Office of Erasmus MC.
