# Supplementary material for: Factors of heavy social media use among 13-year-old adolescents on weekdays and weekends
Source: World J Pediatr. 2023 Feb 20;19(4):378–89. doi: 10.1007/s12519-023-00690-1 (PMC10060361; doi:10.1007/s12519-023-00690-1)
Supplement: Supplementary file 1 — Supplementary file1 (DOCX 22 KB) [file 12519_2023_690_MOESM1_ESM.docx]

Supplementary Table 1 Factors of heavy social media use on a weekday among 13-year-old adolescents

|  | Model 1 | |  | Model 2 | |
| --- | --- | --- | --- | --- | --- |
|  | OR (95%CI) | *P* value |  | OR (95%CI) | *P* value |
| *Demographic variables* |  |  |  |  |  |
| Child age | 1.09 (0.91, 1.31) | 0.331 |  | 0.75 (0.53, 1.07) | 0.111 |
| Child sex |  |  |  |  |  |
| Boy | 1.00 |  |  | 1.00 |  |
| Girl | **1.36 (1.19, 1.55)** | **<0.001** |  | **1.61 (1.32, 1.95)** | **<0.001** |
| Child ethnic background |  |  |  |  |  |
| Dutch | 1.00 |  |  | 1.00 |  |
| Other western | 1.26 (0.99, 1.59) | 0.057 |  | 1.08 (0.78, 1.50) | 0.639 |
| Non-western | **1.32 (1.14, 1.54)** | **<0.001** |  | 1.37 (0.58, 1.76) | 0.107 |
| Family situation |  |  |  |  |  |
| Two-parent family | 1.00 |  |  | 1.00 |  |
| One-parent family | **1.56 (1.32, 1.84)** | **<0.001** |  | **1.42 (1.02, 1.98)** | **0.036** |
| *Socioeconomic position* |  |  |  |  |  |
| Maternal education level |  |  |  |  |  |
| High | 1.00 |  |  | 1.00 |  |
| Middle | **1.37 (1.17, 1.60)** | **<0.001** |  | 0.89 (0.70, 1.14) | 0.351 |
| Low | **1.84 (1.45, 2.34)** | **<0.001** |  | 1.63 (0.64, 2.51) | 0.205 |
| Paternal education level |  |  |  |  |  |
| High | 1.00 |  |  | 1.00 |  |
| Middle | **1.45 (1.22, 1.72)** | **<0.001** |  | 0.97 (0.68, 1.39) | 0.884 |
| Low | **1.64 (1.31, 2.04)** | **<0.001** |  | 1.26 (0.88, 1.79) | 0.206 |
| Maternal employment status |  |  |  |  |  |
| Paid job | 1.00 |  |  | 1.00 |  |
| No paid job | 1.15 (0.97, 1.37) | 0.106 |  | 1.08 (0.81, 1.43) | 0.595 |
| Paternal employment status |  |  |  |  |  |
| Paid job | 1.00 |  |  | 1.00 |  |
| No paid job | 1.03 (0.81, 1.32) | 0.790 |  | 0.77 (0.51, 1.15) | 0.201 |
| Household income |  |  |  |  |  |
| > €3600/month | 1.00 |  |  | 1.00 |  |
| €2000-€3600/month | **1.24 (1.05, 1.46)** | **0.011** |  | 0.92 (0.72, 1.17) | 0.474 |
| < €2000/month | **1.40 (1.16, 1.69)** | **<0.001** |  | 0.89 (0.57, 1.38) | 0.606 |
| *Other screen-based behaviors* |  |  |  |  |  |
| Computer playing | **1.28 (1.20, 1.36)** | **<0.001** |  | **1.33 (1.23, 1.45)** | **<0.001** |
| TV viewing | **1.16 (1.07, 1.25)** | **<0.001** |  | **1.14 (1.03, 1.26)** | **0.011** |
| *Home environment* |  |  |  |  |  |
| Communication |  |  |  |  |  |
| Never/rarely | 1.00 |  |  | 1.00 |  |
| Sometimes/often/always | 1.01 (0.88, 1.16) | 0.896 |  | 0.96 (0.79, 1.15) | 0.634 |
| Supervision |  |  |  |  |  |
| Never/rarely | 1.00 |  |  | 1.00 |  |
| Sometimes/often/always | 0.89 (0.76, 1.05) | 0.157 |  | 0.92 (0.73, 1.16) | 0.481 |
| Restriction |  |  |  |  |  |
| Never/rarely | 1.00 |  |  | 1.00 |  |
| Sometimes/often/always | **0.73 (0.63, 0.85)** | **<0.001** |  | **0.77 (0.63, 0.94)** | **0.011** |

Note: Table is based on non-imputed dataset. Bold print indicates statistical significance at *P* < 0.05.

Values represent odds ratios and 95% confidence intervals derived from (multiple) logistic regression analyses.

Model 1: Each factor was added to the model separately. Model 2: All factors were added to the model.

*OR* odds ratio, *CI* confidence interval

Supplementary Table 2 Factors of heavy social media use on a weekend day among 13-year-old adolescents

|  | Model 1 | |  | Model 2 | |
| --- | --- | --- | --- | --- | --- |
|  | OR (95%CI) | *P* value |  | OR (95%CI) | *P* value |
| *Demographic variables* |  |  |  |  |  |
| Child age | 1.19 (0.99, 1.43) | 0.064 |  | 0.89 (0.63, 1.25) | 0.486 |
| Child sex |  |  |  |  |  |
| Boy | 1.00 |  |  | 1.00 |  |
| Girl | **1.36 (1.19, 1.55)** | **<0.001** |  | **1.48 (1.20, 1.82)** | **<0.001** |
| Child ethnic background |  |  |  |  |  |
| Dutch | 1.00 |  |  | 1.00 |  |
| Other western | 1.16 (0.91, 1.47) | 0.228 |  | 0.89 (0.65, 1.23) | 0.486 |
| Non-western | **1.34 (1.15, 1.56)** | **<0.001** |  | 1.45 (0.98, 2.14) | 0.062 |
| Family situation |  |  |  |  |  |
| Two-parent family | 1.00 |  |  | 1.00 |  |
| One-parent family | **1.56 (1.31, 1.86)** | **<0.001** |  | **1.66 (1.18, 2.35)** | **0.004** |
| *Socioeconomic position* |  |  |  |  |  |
| Maternal education level |  |  |  |  |  |
| High | 1.00 |  |  | 1.00 |  |
| Middle | **1.54 (1.31, 1.80)** | **<0.001** |  | 1.19 (0.93, 1.52) | 0.164 |
| Low | **1.60 (1.24, 2.05)** | **<0.001** |  | 1.06 (0.66, 1.68) | 0.820 |
| Paternal education level |  |  |  |  |  |
| High | 1.00 |  |  | 1.00 |  |
| Middle | **1.70 (1.43, 2.02)** | **<0.001** |  | 1.45 (0.99, 2.11) | 0.054 |
| Low | **1.90 (1.51, 2.39)** | **<0.001** |  | **1.69 (1.31, 2.17)** | **<0.001** |
| Maternal employment status |  |  |  |  |  |
| Paid job | 1.00 |  |  | 1.00 |  |
| No paid job | 1.19 (1.00, 1.42) | 0.052 |  | 1.23 (0.98, 1.54) | 0.079 |
| Paternal employment status |  |  |  |  |  |
| Paid job | 1.00 |  |  | 1.00 |  |
| No paid job | 1.03 (0.81, 1.32) | 0.793 |  | 1.03 (0.69, 1.55) | 0.891 |
| Household income |  |  |  |  |  |
| > €3600/month | 1.00 |  |  | 1.00 |  |
| €2000-€3600/month | 1.16 (0.96, 1.40) | 0.129 |  | 1.16 (0.76, 1.79) | 0.493 |
| < €2000/month | **1.19 (1.01, 1.41)** | **0.035** |  | **1.65 (1.05, 2.60)** | **0.031** |
| *Other screen-based behaviors* |  |  |  |  |  |
| Computer playing | **1.35 (1.28, 1.42)** | **<0.001** |  | **1.42 (1.33, 1.53)** | **<0.001** |
| TV viewing | **1.10 (1.04, 1.17)** | **0.002** |  | **1.13 (1.05, 1.23)** | **0.002** |
| *Home environment* |  |  |  |  |  |
| Communication |  |  |  |  |  |
| Never/rarely | 1.00 |  |  | 1.00 |  |
| Sometimes/often/always | 1.12 (0.98, 1.29) | 0.089 |  | 1.14 (0.94, 1.37) | 0.174 |
| Supervision |  |  |  |  |  |
| Never/rarely | 1.00 |  |  | 1.00 |  |
| Sometimes/often/always | **0.80 (0.69, 0.93)** | **0.004** |  | **0.72 (0.54, 0.96)** | **0.026** |
| Restriction |  |  |  |  |  |
| Never/rarely | 1.00 |  |  | 1.00 |  |
| Sometimes/often/always | 0.87 (0.75, 1.00) | 0.054 |  | 0.95 (0.77, 1.16) | 0.591 |

Note: Table is based on non-imputed dataset. Bold print indicates statistical significance at *P* < 0.05.

Values represent odds ratios and 95% confidence intervals derived from (multiple) logistic regression analyses.

Model 1: Each factor was added to the model separately. Model 2: All factors were added to the model.

*OR* odds ratio, *CI* confidence interval

Supplementary Table 3 The contribution by each factor to the model (on a weekday)

|  | χ^2^ | Nagelkerke R Squared |
| --- | --- | --- |
| *Demographic variables* |  |  |
| Child age | 0.939 | 0.000 |
| Child sex | 18.702 | 0.007 |
| Child ethnic background | 34.081 | 0.013 |
| Family situation | 52.245 | 0.020 |
| *Socioeconomic position* |  |  |
| Maternal education level | 60.340 | 0.025 |
| Paternal education level | 61.586 | 0.028 |
| Maternal employment status | 66.143 | 0.030 |
| Paternal employment status | 61.534 | 0.029 |
| Household income | 62.098 | 0.032 |
| *Other screen time behaviors* |  |  |
| Computer playing | 101.675 | 0.062 |
| TV viewing | 104.469 | 0.064 |
| *Home environment* |  |  |
| Communication | 98.623 | 0.062 |
| Supervision | 96.318 | 0.061 |
| Restriction | 102.241 | 0.066 |

Note: Table is based on imputed dataset.

χ^2^ was obtained by the Omnibus Test. Each factor was forward entry to model (i.e. step1: child age; step 2: child age + child sex; step3: child age + child sex + child ethnic background).

Nagelkerke R Squared was obtanied by the Model Summary.

Supplementary Table 4 The contribution by each factor to the model (on a weekend day)

|  | χ^2^ | Nagelkerke R Squared |
| --- | --- | --- |
| *Demographic variables* |  |  |
| Child age | 3.513 | 0.001 |
| Child sex | 18.702 | 0.007 |
| Child ethnic background | 13.595 | 0.005 |
| Family situation | 35.129 | 0.013 |
| *Socioeconomic position* |  |  |
| Maternal education level | 48.318 | 0.020 |
| Paternal education level | 60.461 | 0.027 |
| Maternal employment status | 59.393 | 0.027 |
| Paternal employment status | 67.058 | 0.031 |
| Household income | 73.595 | 0.037 |
| *Other screen time behaviors* |  |  |
| Computer playing | 187.509 | 0.111 |
| TV viewing | 192.208 | 0.115 |
| *Home environment* |  |  |
| Communication | 167.883 | 0.103 |
| Supervision | 173.443 | 0.107 |
| Restriction | 169.216 | 0.106 |

Note: Table is based on imputed dataset.

χ^2^ was obtained by the Omnibus Test. Each factor was forward entry to model (i.e. step1: child age; step 2: child age + child sex; step3: child age + child sex + child ethnic background).

Nagelkerke R Squared was obtanied by the Model Summary.
